# The complete mitochondrial genome of blue-lined octopus *Hapalochlaena fasciata* (Hoyle, 1886) (Octopodiformes; Octopoda; Octopodidae)

**DOI:** 10.1080/23802359.2020.1778557

**Published:** 2020-09-08

**Authors:** Hye Seon Kim, Keun-Yong Kim, Il-Hun Kim, Dongwoo Yang, Haryun Kim

**Affiliations:** a National Marine Biodiversity Institute of Korea, Seocheon, Republic of Korea; b AquaGenTech Co., Ltd, Busan, Republic of Korea

**Keywords:** Blue-lined octopus, *Hapalochlaena fasciata*, mitochondrial genome, phylogeny

## Abstract

The complete mitochondrial genome of the highly venomous blue-lined octopus, *Hapalochlaena fasciata* (Hoyle, 1886), was analyzed by the primer walking method. Its mitogenome was 15,479 bp in total length, comprising 13 protein-coding genes, 2 ribosomal RNA genes, and 23 transfer RNA genes. In the phylogenetic tree, the gene content and order were congruent with those of typical octopodiform species. The mitogenomic sequence presented could be very useful as the first record of the complete mitogenome for the genus *Hapalochlaena*.

The genus *Hapalochlaena* are only octopuses known to be capable of causing toxic morbidity or mortality in humans (White and Meier 1995). Among them, the shallow-water benthic octopus *Hapalochlaena fasciata*, which is also called as the blue-lined octopus, is most commonly found around intertidal rocky shores and coastal waters between Australia and the Pacific Ocean north to Japan (Williamson et al. 1996; Kohtsuka 2006; Kim et al. 2012). In Korea, the appearance of *H. fasciata* is increasing every year (Kim et al. 2018), but there are very little scientific records. In this study, we reported the complete mitochondrial genome (mitogenome) of the highly venomous blue-lined octopus, *H. fasciata* from the southern coastal waters, Korea.

A specimen of *H. fasciata* was collected in the southern coastal water of Korea, at 15 Nov. 2019 (34°26′17.93ʺN, 127°51′32.36ʺE) and deposited at the National Marine Biodiversity Institute of Korea (voucher no. MABIK MO00176321) after ethanol fixation. Its genomic DNA was extracted from a tissue around the mouth with DNeasy Blood & Tissue Kit (Qiagen, Hilden, Germany). Its complete mitogenome was amplified through two independent and overlapping PCR runs, and the PCR products were sequenced using 25 sequencing primers. The complete mitogenomic sequence was deposited in the GenBank under the accession number MT497543.

All mitogenomic sequences of the species belonging to the superorder Octopodiformes were retrieved from GenBank in NCBI (http://www.ncbi.nlm.nih.gov/). They were aligned together with that of *H. fasciata* in this study and refined manually. For the phylogenetic analysis, the nucleotide matrix of 13 protein-coding genes was created and divided into three partitions, consisting of 3706 bp each for the first, second, and third bases of each codon triplet, respectively. A phylogenetic tree was reconstructed using RAxML 7.0.4 (Stamatakis 2006) for maximum-likelihood (ML) analysis.

The complete mitogenome of *H. fasciata* was a circular molecule of 15,479 bp in total length, consisting of 13 protein-coding genes, 2 ribosomal RNA genes, and 22 transfer RNA genes, of which gene content is identical those of typical octopuses. Its gene order was also identical to those of typical octopuses, but different from those of decapodiform species.

With the mitogenomic sequence of *H. fasciata*, a phylogenetic tree was reconstructed by the ML method, using the nucleotide sequence matrix from 13 protein-coding genes (Figure 1). In the resulting tree, octopodiform species formed the monophyletic group with 100% bootstrap value with respect to the two decapodiform outgroups. Among the lineage, *H. fasciata* in this study consistently emerged among *Amphioctopus* species with close phylogenetic relationship to *Amphioctopus aegina*, *A. marginatus*, and *A. neglectus* with 85% bootstrap value.

**Figure 1.**
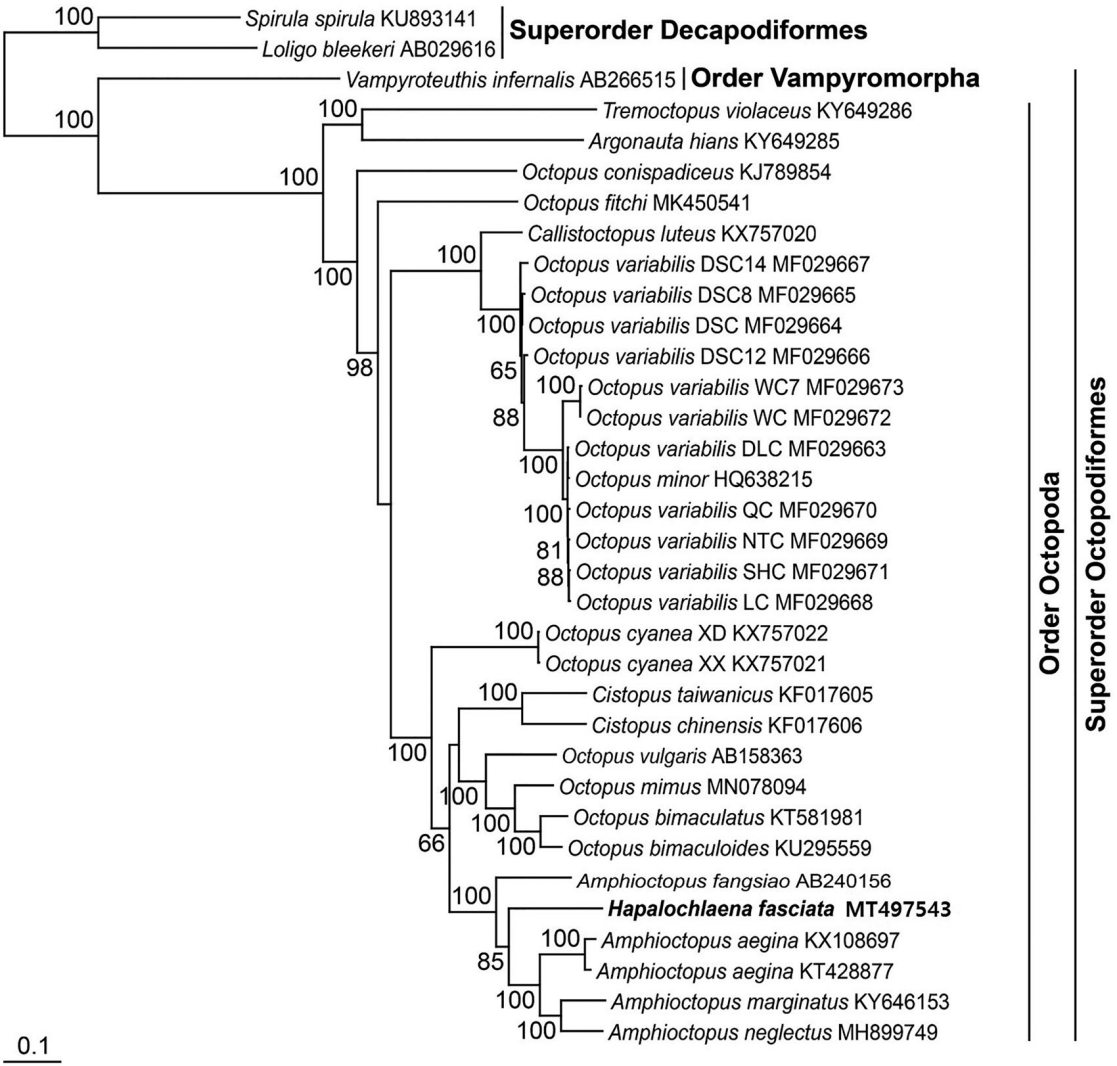
Maximum-likelihood (ML) phylogeny based on the complete mitochondrial genomes from the octopuses belonging to the superorder Octopodiformes. The nucleotide sequence matrix included the three codon positions of the 13 protein-coding genes. A bootstrap value above 50% in the ML analysis is indicated at each node. *Hapalochlaena fasciata* analyzed in this study is shown in bold.

In this study, the complete mitogenomic sequence of the genus *Hapalochlaena* was analyzed for the first time, and the phylogenetic tree among octopuses was reconstructed with reference to their mitogenomic sequence data. Our data will provide baseline data for the molecular identification, geographical distribution and dispersal, population genetics, and early warning for the highly venomous marine cephalopod species, *H. fasciata* in the Korean coastal waters.

## Data Availability

The data that support the findings of this study are openly available in GenBank of NCBI at https://www.ncbi.nlm.nih.gov, reference number MT497543.
